# Generation and characterization of a Myh6-driven Cre knockin mouse line

**DOI:** 10.1007/s11248-021-00285-4

**Published:** 2021-09-20

**Authors:** Xinyan Huang, Lei Yan, Shan Kou, Jufeng Meng, Zhengkai Lu, Chao-Po Lin, Chen Liu, Hui Zhang

**Affiliations:** 1 School of Life Science and Technology, ShanghaiTech University, 393 Middle Huaxia Road, Pudong, Shanghai, 201210 China; 2 Department of Cardiac Surgery, Zhongshan Hospital, Fudan University, 1409 Xietu Road, Xuhui, Shanghai, 200032 China; 3 University of Chinese Academy of Sciences, Beijing, 100049 China

**Keywords:** Cre-loxp, Mouse model, Development, Desmoplakin

## Abstract

Gene deletion by the Cre-Loxp system has facilitated functional studies of many critical genes in mice, offering important insights and allowing deeper understanding on the mechanisms underlying organ development and diseases, such as heart development and diseases. In this study, we generated a *Myh6-Cre* knockin mouse model by inserting the IRES-Cre-wpre-polyA cassette between the translational stop codon and the 3′ untranslated region of the endogenous *Myh6* gene. By crossing knockin mice with the *Rosa26* reporter lines, we found that *Myh6-Cre* targeted cardiomyocytes at the embryonic and postnatal stages. In addition, we were able to inactivate the desmosome gene Desmoplakin (Dsp) by breeding *Myh6-Cre* mice with a conditional *Dsp*^*flox*^ knockout mouse line, which resulted in embryonic lethality during the mid-term pregnancy. These results suggest that the new *Myh6-Cre* mouse line can serve as a robust tool to dissect the distinct roles of genes involved in heart development and function.

**Supplementary Information:**

The online version contains supplementary material available at 10.1007/s11248-021-00285-4.

## Introduction

Congenital heart disease is the most common birth defect in humans and the leading cause of death in the first year of life (Hoffman 1995; Wolf and Basson 2010; Bruneau and Srivastava 2014; Gelb and Chung 2014). Gene deletion by the Cre-Loxp system has facilitated causative studies of many genes in mice that are essential for heart development and function. A variety of transgenic constitutive or inducible Cre mouse tools have been generated and employed in order to manipulate the expression of target genes in cardiomyocytes. However, transgenes may not always correctly reflect the expression of endogenous genes (Laboulaye et al. 2018), and the copy number of insertional transgenes may be reduced during passages which directly influences Cre expression levels (Davis et al. 2012). For the inducible CreER or MerCreMer (Cre recombinase fused to two mutated estrogen receptor ligand binding domains), regardless of being transgenic or knockin mouse lines, the inducible Cre is not able to mediate complete excision, and the administration of tamoxifen is toxic to embryos and could easily lead to abortion before sample collection. Furthermore, some transgenic inducible Cre mice displayed cardiac functional defects after tamoxifen treatment (Buerger et al. 2006; Hall et al. 2011; Koitabashi et al. 2009; Hougen et al. 2010; Lexow et al. 2013; Bersell et al. 2013; Molkentin and Robbins 2009), which may lead to the misinterpretation of data in different studies.

*Myh6*, also known as *αMHC*, *Myhc* and *Myhca*, encodes the cardiac muscle specific protein, alpha-myosin heavy chain, which is dynamically expressed in cardiomyocytes and with significant importance for heart development (Carniel et al. 2005; Ching et al. 2005; Posch et al. 2011; Ng et al. 1991). Mutations in MYH7 in humans cause dilated and hypertrophic cardiomyopathy (Carniel et al. 2005; Richard et al. 2003). MYH6 is expressed at low levels in adult human cardiomyocytes and the role of genetic variants in MYH6 in human diseases is uncertain. In this study, we created a *Myh6-Cre* knockin mouse line by inserting the IRES-Cre-wpre-polyA cassette between the translational stop codon and the 3′ untranslated region (UTR) of the endogenous *Myh6* gene. By immunostaining experiments, we found that *Myh6-Cre* targeted cardiomyocytes during fetal and postnatal stages. In addition, we were able to efficiently delete the desmosome gene Desmoplakin (Dsp) from the mouse heart using *Myh6-Cre*, which resulted in embryonic lethality during mid-term pregnancy. Our results showed that *Myh6-Cre* is a useful genetic tool that enables the deletion of target genes in cardiomyocytes.

## Materials and methods

### Animals

All mouse studies were performed in accordance with the guidelines provided by the institutional Animal Care and Use Committee at ShanghaiTech University. All mice were maintained in specific pathogen-free conditions. Mice were bred with a normal diet and maintained on a C57BL6/J/ICR background. Both male and female animals were used in the analyses. *R26-tdTomato* and *Dsp*^*flox*^ mice have been previously reported (Madisen et al. 2010; Vasioukhin et al. 2001). The *R26-tdTomato* mouse line was kindly provided by the Shanghai Model Organisms Center, INC. The *Dsp*^*flox*^ mouse line was purchased from the Jackson Laboratory. For generation of the *Myh6-Cre* mouse line, the CRISPR/Cas9 technology was used to insert the internal ribosome entry site (IRES)-Cre cassette, the woodchuck hepatitis virus posttranscriptional regulatory element (wpre), and a polyA sequence between the translational stop codon and the 3′ UTR of the endogenous *Myh6* gene. The *Myh6-Cre* mouse line was generated by the Shanghai Model Organisms Center, INC. This newly generated mouse line is available from the corresponding author with a completed material transfer agreement.

### Genotyping

Genomic DNA was extracted from either the embryonic yolk sac or the mouse tail. The harvested tissues were lysed through incubation with proteinase K for a total of 12 h at a temperature of 55 °C, followed by centrifugation for 8 min. This process allowed us to obtain a supernatant containing the genomic DNA, which was precipitated by adding isopropanol. All embryos and mice used in our experiments were genotyped with specific primers that distinguished the knockin from the wild-type alleles. The genotyping primers used in the *Myh6-Cre* mouse line were: 5′-TTCCCAAGGGCATTTTATTAG-3′ and 5′-CTTTGGGCTTGGCATCATCTGGT-3′ (wild type allele); 5′-CAGAAGATGCACGACGAG-3′ and 5′-CAGCCCCTTGTTGAATACG-3′ (knock-in allele).

### Immunostaining

We performed immunofluorescence staining according to previously described protocols (Huang et al. 2019). Briefly, mouse embryos or hearts were collected and fixed in 4% paraformaldehyde (PFA) for a total of 30 min to 1 h, depending on the age of the tissues. After three consecutive washes in PBS, mouse embryos or hearts were photographed using fluorescence microscopy (Olympus, MVX10). The tissues were then dehydrated in 30% sucrose and embedded in optimum cutting temperature (OCT) compound (Sakura). Cryosections collected with 9-μm thickness were air-dried and then blocked with blocking buffer (5% donkey serum, 0.1% Triton X-100 in PBS). Both of these steps were performed for 30 min each at room temperature. Primary antibodies were incubated overnight at 4 °C. The following primary antibodies were used: tdTomato (Rockland, 600–4,010,379, 1:1000; Sicgen, AB8181-200, 1:1000), PDGFRa (R&D, AF1062, 1:500), PDGFRb (eBioscience, 14-1402-82, 1:500), aSMA (Sigma, F3777, 1:400), CDH5 (R&D, AF1002, 1:200), TNNI3 (Abcam, ab56357, 1:200), Cre (Millipore, MAB3120, 1:200), MYH6 (Invitrogen, PA5-97,224,1:200) and WT1 (Abcam, ab89901, 1:100). Signals were developed with Alexa fluorescence antibodies (Invitrogen). The images obtained were acquired using a confocal microscope (Nikon A1R).

### Real-time quantitative PCR

Mouse embryos at embryonic day 10.5 or adult hearts at postnatal 8 weeks were harvested. The hearts or non-cardiac tissues were treated with Trizol in order to extract RNA in accordance with the manufacturer’s instructions (Invitrogen). We converted the RNA to cDNA using the Prime Script RT kit (Vazyme). We used the SYBR Green qPCR master mix (Vazyme) and amplified the cDNA on a StepOnePlusTM real-time PCR system (Applied Biosystems). The primers used to detect the mRNA levels of the *Dsp*, *Myh6* and *Gapdh* are listed below. *Dsp*: 5′-AAACCGGCACCATGTCTAGA-3′ and 5′- CTCCGAATTTCAGTTCCGGC-3′; *Myh6*: 5′-CAATGCAGAGTCGGTGAAGG-3′ and 5′-CCTCTGTCTGGTAGGTGAGC-3′; *Gapdh*: 5′-TTGTCTCCTGCGACTTCAAC-3′ and 5′-GTCATACCAGGAAATGAGCTTG-3′.

### Hematoxylin and Eosin staining

Mouse hearts at postnatal 8 weeks were fixed in 4% PFA at 4 °C for 1 h and then dehydrated in 30% sucrose overnight. The next day, the hearts were processed into OCT-embedded serial sections. Slides were incubated in hematoxylin solution for 8–10 min and then rinsed in running tap water. The slides were then treated with 1% concentrated hydrochloric acid in 70% ethanol for 1.5 min and rinsed in running tap water. After soaking in PBS for 3 min and washed by running tap water, the slides were stained with eosin solution for 3–5 min followed by dehydration in ethanol and xylene. Images were acquired by stereomicroscope (Olympus MVX10).

### Echocardiographic assessment

Adult mice at postnatal 8 weeks or 4 months were anesthetized with inhalation of isoflurane (2.5%-3%) and heart rate was maintained at 400–500 bpm. The chest was shaved and further cleaned with hair removal gel cream. Mice were placed on a warm board in the supine position with the limbs taped onto the metal electrocardiographic leads. Acoustic gel was applied to the thorax surface as coupling medium. Using a VisualSonics Vevo 2100 system and a 30-MHz microvisualization scan head probe, M-mode images and real-time B-mode cine loops of the left ventricle were acquired for cardiac structure and function assessment. Using the long-axis view, left ventricular end-systolic volume and end-diastolic volume, as well as the ejection fraction were calculated by identifying frames with the maximal and minimal cross-sectional area and width. All data were presented as mean values ± SEM. The echocardiographers were blinded to animal group assignments.

### Cardiomyocyte isolation

*Myh6-Cre/* + *;R26-tdTomato/* + mice at postnatal 8 weeks were intraperitoneally injected with about 150 USP units heparin 15 min in advance. Then mice were intraperitoneally injected with 10% chloral hydrate. The dissected hearts were perfused with modified Tyrode’s solution (MTS) for 5 min. Later, the hearts were perfused with MTS containing 1 mg/ml collagenase II and 0.08 mg/ml Protease XIV. The digested hearts were shredded with forceps and filtered through a 70 µm strainer. The filtered cells were centrifuged at 20 × g for 3 min at 4 °C and most of the cardiomyocytes were pelleted. The pellets were re-suspended in MTS with 0.5% BSA and then centrifuged at 100 × g for 5 min at 4 °C in Percoll to remove the dead cells. The cardiomyocytes were re-suspended in MTS with 0.5% BSA and implanted in 24-well plates. Images were taken under a stereo microscope (Olympus MVX10).

### Western blot

The hearts at postnatal 8 weeks were dissected and washed in PBS buffer. Then about 15 mg tissues were lysed in 300 µL RIPA lysis buffer and incubated on ice for 30 min. All protein samples were mixed with 5 × loading buffer and boiled at 95 °C for 5 min. The lysates were analyzed by SDS/PAGE and transferred onto the polyvinylidene fluoride membrane. Membranes were blocked for 1 h at room temperature by using 5% BSA-TBST solution. Then the membranes were incubated with primary antibodies Anti-MYH6 (Invitrogen, PA5-97,224, 1:1000), Anti-Cre recombinase (Millipore, MAB3120, 1:1000) at 4 °C overnight. The next day, after washing primary antibodies with TBST, membranes were incubated with HRP-conjugated secondary antibodies at room temperature for 1 h. HRP-anti-β-Actin (ZENBIO, 700068, 1:10000) primary antibodies were just incubated at room temperature for 1 h. Signals were revealed with the enhanced chemiluminescence kit.

### Statistics analysis

All data were representative of at least three independent experiments and presented as mean values ± SEM. Data of the two groups were analyzed using an unpaired Student’s t-test, and comparison between more than two groups was performed using analysis of variance (ANOVA), followed by Tukey’s multiple comparison test. *p* < 0.05 indicated statistical significance.

## Results

### Generation of *Myh6*-Cre mice

**Fig. 1:**
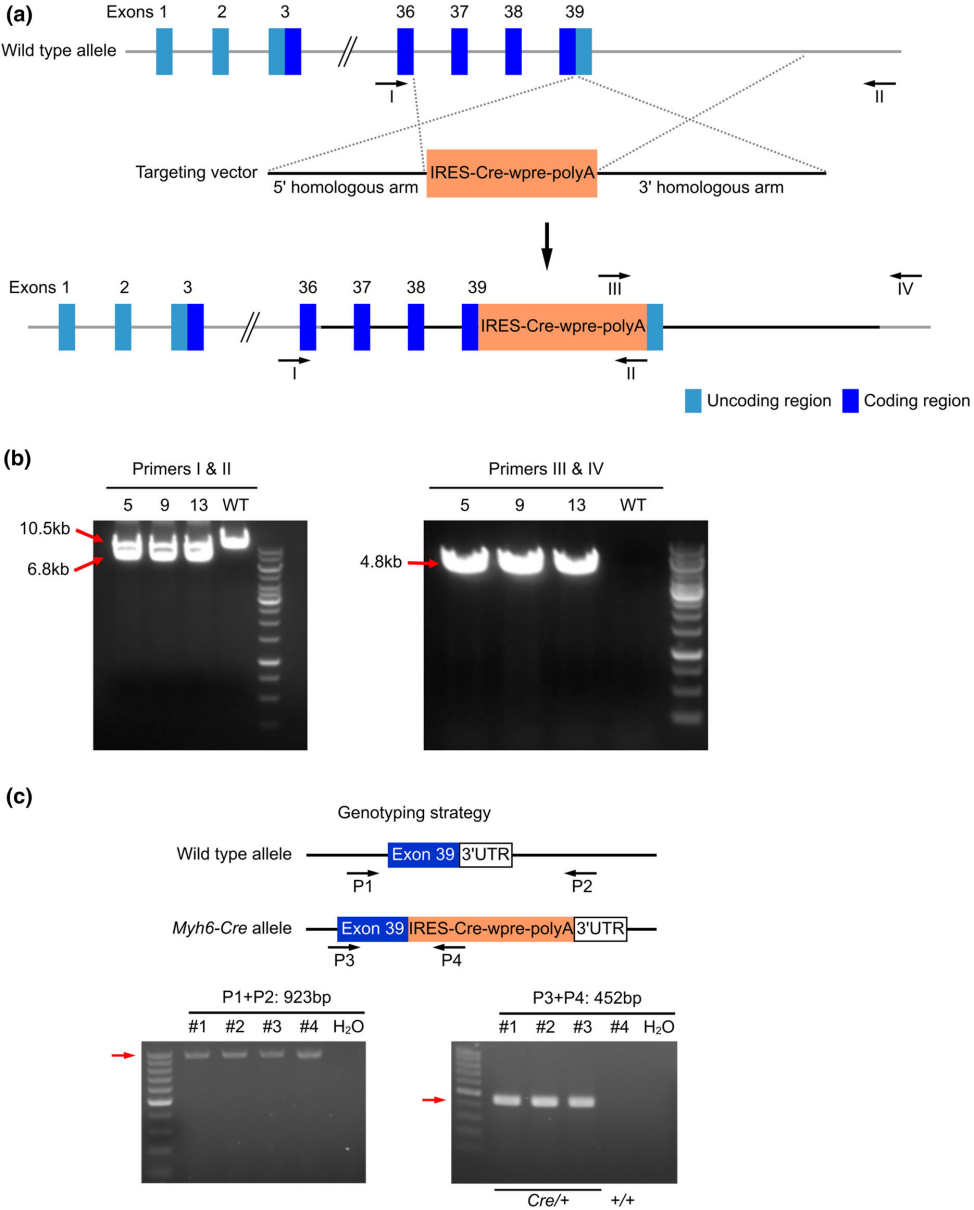
Strategy for generating *Myh6-Cre* mice. **a** The strategy for generating *Myh6-Cre mice*. The IRES-Cre-wpre-polyA cassette was inserted between the translational stop codon and the 3'UTR. **b** PCR results show that homologous recombination was detected in the F0 generation mice. **c** Locations of the genotyping primers and an example of genotyping result showing the PCR products

*Myh6* mutations lead to dilated and hypertrophic cardiomyopathy, as well as atrial septal defect (Posch et al. 2011; Ching et al. 2005; Carniel et al. 2005). In order to avoid mutating or disrupting *Myh6*, we generated a knockin mouse line *Myh6-Cre* using CRISPR/Cas9 to insert the IRES-Cre-wpre-polyA cassette between the translational stop codon and the 3′ UTR of the endogenous *Myh6* gene (Fig. 1a). Specifically, we injected the Cas9 mRNA, gRNA (5′-acagcgagggtctgctggag-3′), and the donor targeting vector, which contains 5′ homologous arm (2.5 kb), IRES-Cre-wpre-poly A cassette, and 3′ homologous arm (4.0 kb), into C57BL6/J zygotes, which were subsequently transferred into pseudo-pregnant mice. With 21 offspring of F0 generation obtained, we designed four primers (Primers I and II to amplify the wild type allele [10.5 kb] or the recombined 5′ arm [6.8 kb]; Primers III and IV to amplify the recombined 3′ arm [4.8 kb]) to screen the F0 mice, and finally found 3 founders with successful homologous recombination (Fig. 1a, b). We next sequenced the PCR products to further confirm the correct recombination, and then crossed the 3 founders with wild type mice to get F1 generation.

To optimize the genotyping protocol for *Myh6-Cre* knockin, we re-designed four PCR primers (P1 and P2 to amplify the wild type allele [923 bp] and P3 and P4 to amplify the inserted site [452 bp]), which were located outside or inside of the IRES-Cre-wpre-polyA cassette sequence (Fig. 1c). Genotyping by PCR using the forward and reverse primers showed that the IRES-Cre-wpre-polyA cassette was successfully inserted into the *Myh6* locus (Fig. 1c).

### *Myh6-Cre* labels cardiomyocytes from embryonic stage to adulthood

To characterize the *Myh6-Cre* mouse line, we subsequently crossed the *Myh6-Cre/* + mice with a reporter line containing a stop cassette flanked by loxp sites upstream of tdTomato at the *Rosa26* locus (*Rosa26-loxp-stop-loxp-tdTomato*, or *R26-tdTomato*)(Madisen et al. 2010). This crossing allowed us to generate a *Myh6-Cre/* + *;R26-tdTomato/* + mouse line. Importantly, the loxp-flanked stop cassette, which prevents the transcription of the downstream tdTomato, was thus deleted in the Cre-expressing cells, leading to the expression of tdTomato.

**Fig. 2:**
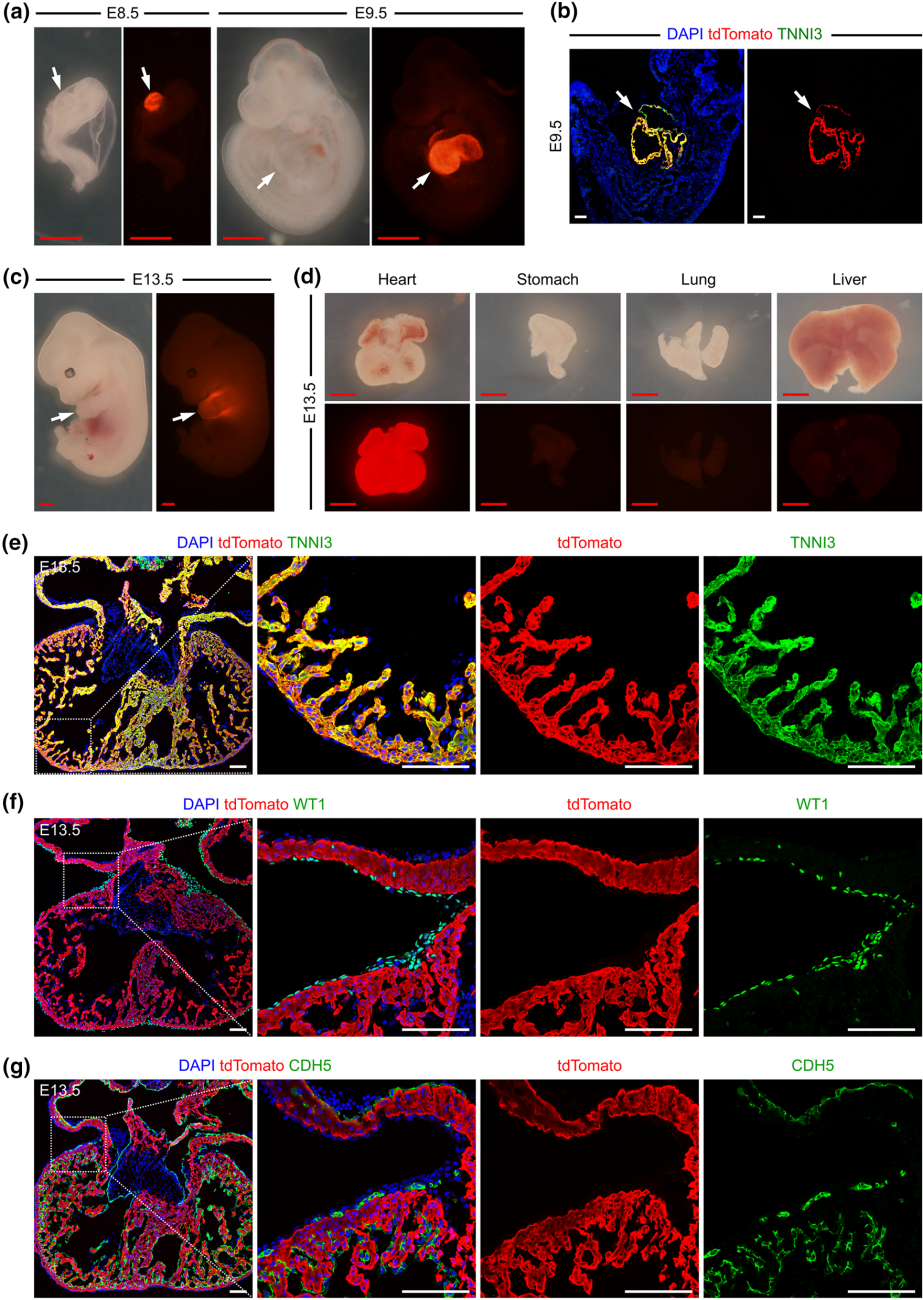
*Myh6-Cre* labels cardiomyocytes in embryonic hearts. (**a**, **c**) Whole-mount view of the *Myh6-Cre/* + *;R26-tdTomato/* + embryos at E8.5 and E9.5. **b** Immunostaining for tdTomato and TNNI3 on embryonic sections at E9.5. The arrows in (**a**–**c**) indicate hearts with enriched tdTomato signals. **d** Whole-mount view of the organs from *Myh6-Cre/* + *;R26-tdTomato/* + *mice* at E13.5. (**e**–**g**) Immunostaining for tdTomato and TNNI3, WT1 or CDH5 on heart sections from *Myh6-Cre/* + *;R26-tdTomato/* + mice at E13.5. Boxed regions are magnified on the right. 4 embryos were examined for each embryonic stage. Red scale bars, 1 mm; White scale bars, 100 µm

We examined the *Myh6-Cre/* + *;R26-tdTomato/* + embryos at different time points. At embryonic day (E) 8.5, we specifically detected tdTomato signals in the heart tubes (Fig. 2a). At E9.5, we found similar results that tdTomato was exclusively expressed in the hearts (Fig. 2a). Immunostaining for tdTomato and cardiomyocyte marker TNNI3 showed that most cardiomyocytes were labelled by tdTomato at E9.5 (Fig. 2b). We then analyzed the embryos at E13.5. tdTomato signals were robustly detected in the heart, but not in other organs, such as the stomach, lung and liver (Fig. 2c, d). Immunostaining for tdTomato and TNNI3 confirmed that the majority of cardiomyocytes were tdTomato^+^ at E13.5 (Fig. 2e). Moreover, co-staining assays of different heart sections with tdTomato and either WT1 or CDH5 antibodies showed that tdTomato did not target any epicardial or endothelial cells at E13.5 (Fig. 2f, g). We also stained the *Myh6-Cre/* + embryonic hearts with TNNI3, MYH6 and Cre antibodies and found co-localization of Cre and MYH6 in TNNI3^+^ cardiomyocytes (Supplementary Fig. S1), suggesting that Cre activity is specifically localized to MYH6^+^ cardiomyocytes with high fidelity in *Myh6-Cre/* + embryonic hearts.

**Fig. 3:**
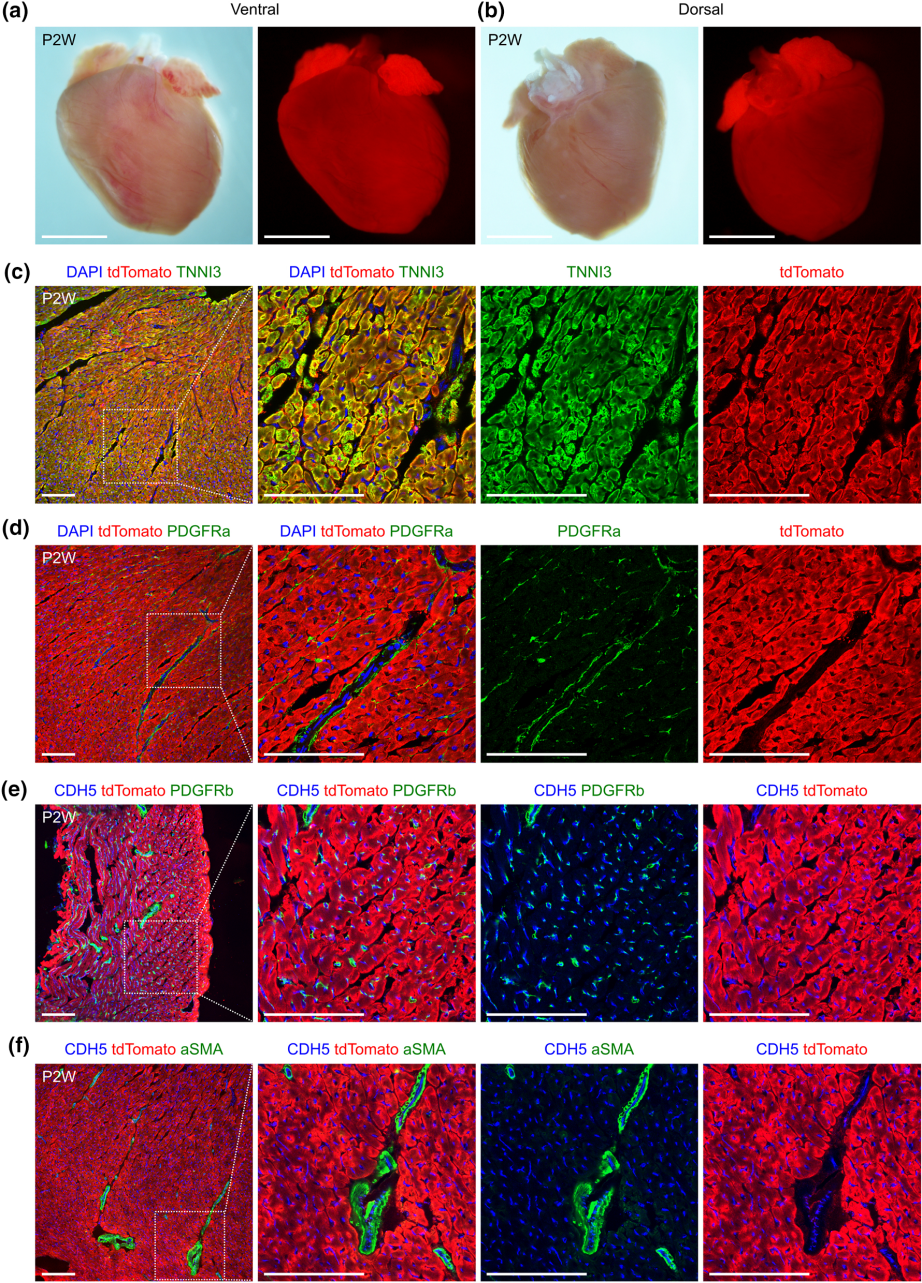
*Myh6-Cre* labels cardiomyocytes in infant mice. (**a**, **b**) Whole-mount view of the hearts from *Myh6-Cre/* + *;R26-tdTomato/* + mice at P2W. Scale bars, 5 mm. (**c**, **d**) Immunostaining for tdTomato and TNNI3 or PDGFRa on heart sections from *Myh6-Cre/* + *;R26-tdTomato/* + *mice* at P2W. (**e**, **f**) Immunostaining for tdTomato, CDH5 and PDGFRb or aSMA on heart sections from *Myh6-Cre/* + *;R26-tdTomato/* + mice at P2W. Boxed regions in (**c**–**f**) are magnified on the right. Scale bars in (**c**–**f**), 100 µm. Each picture is representative of 3 individual mouse samples

Next, we proceeded to analyze the *Myh6-Cre/* + *;R26-tdTomato/* + mice at postnatal stages. We harvested the hearts at postnatal 2 weeks (P2W) and were able to detect significant tdTomato signals in the whole-mount views (Fig. 3a, b). Similarly, co-staining for tdTomato and TNNI3 on heart sections also demonstrated that the majority of cardiomyocytes were tdTomato^+^ at P2W (Fig. 3c). PDGFRa has been previously used as a marker for cardiac fibroblasts (Kaur et al. 2016; Huang et al. 2019; Feng et al. 2019). Hence, in order to examine whether the lineage tracer marks cardiac fibroblasts at P2W, we stained the heart sections with tdTomato and PDGFRa antibodies. No tdTomato-labelled PDGFRa^+^ cells were found (Fig. 3d), suggesting that cardiac fibroblasts were not targeted by tdTomato. Additionally, we stained the hearts with antibodies against tdTomato, CDH5 and PDGFRb, a gene which was previously reported as a coronary vascular pericyte marker (Chen et al. 2016; Huang et al. 2019). Similarly, we could not find any tdTomato-labelled coronary endothelial cells or pericytes at P2W (Fig. 3e). Finally, we were not able to identify any tdTomato-labelled smooth muscle cells (SMCs) (aSMA^+^) in hearts at P2W (Fig. 3f). Taken together, our results provide strong evidence that *Myh6-Cre/* + *;R26-tdTomato/* + specifically targets cardiomyocytes in early mouse infants.

**Fig. 4:**
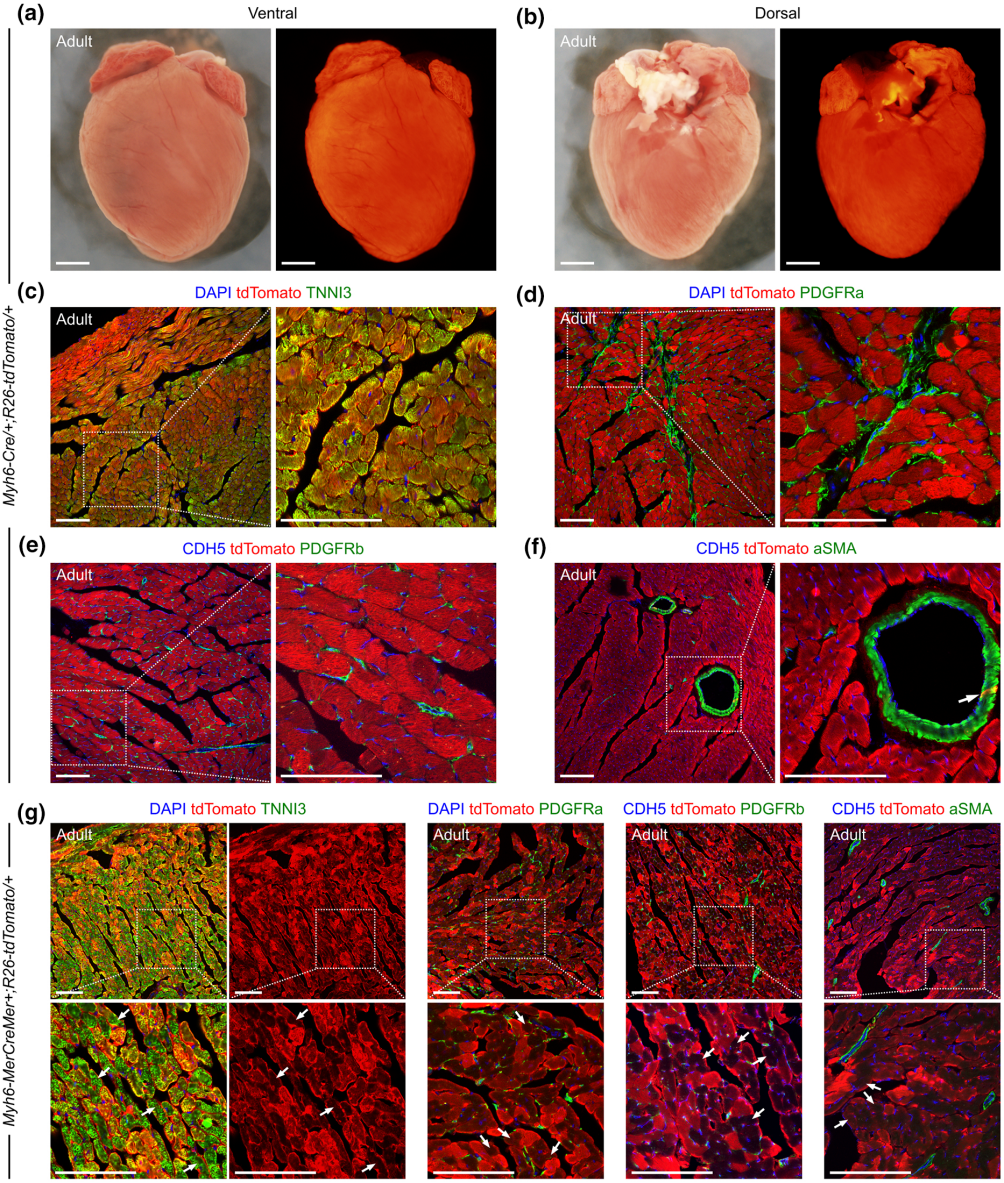
*Myh6-Cre* labels cardiomyocytes and a small portion of smooth muscle cells in adult hearts. (**a**, **b**) Whole-mount view of the hearts from adult *Myh6-Cre/* + *;R26-tdTomato/* + mice. Scale bars, 2 mm. (**c**, **d**) Immunostaining for tdTomato and TNNI3 or PDGFRa on heart sections from adult *Myh6-Cre/* + *;R26-tdTomato/* + mice. (**e**, **f**) Immunostaining for tdTomato, CDH5 and PDGFRb or aSMA on heart sections from adult *Myh6-Cre/* + *;R26-tdTomato/* + mice. The arrows in (**f**) indicate tdTomato-labelled smooth muscle cell. **g** Immunostaining on heart sections from adult *Myh6-MerCreMer* + *;R26-tdTomato/* + mice, which were administered with tamoxifen 48 h before harvest. The arrows in (**g**) indicate cardiomyocytes with weak tdTomato signal in the cytoplasm. Boxed regions are magnified as indicated. Scale bars in (**c**–**g**), 100 µm. Each picture is representative of 3 individual mouse samples

We subsequently examined the hearts from adult *Myh6-Cre/* + *;R26-tdTomato/* + mice at P8W. Similar to P2W, we identified strong tdTomato signals in the whole-mount view of the hearts (Fig. 4a, b), and found that the significant majority of cardiomyocytes were targeted by tdTomato by staining on heart sections (Fig. 4c). We also confirmed the tdTomato expression in the isolated cardiomyocytes from P8W hearts (Supplementary Fig. S2). Moreover, our observations confirmed that no PDGFRa^+^ fibroblasts, CDH5^+^ coronary endothelial cells, or PDGFRb^+^ vascular pericytes were marked by tdTomato in the adult hearts (Fig. 4d, e). However, we did find tdTomato expression in a few coronary vascular SMCs (Fig. 4f). Microscopic quantification shows that 9.42 ± 2.84% of SMCs are labeled in the adult *Myh6-Cre/* + *;R26-tdTomato/* + mouse hearts. Collectively, these observations demonstrate that *Myh6-Cre/* + *;R26-tdTomato/* + is also able to efficiently target cardiomyocytes in adult hearts.

To compare the labeling specificity *Myh6-Cre* with other similar Cre strains, we employed the inducible transgenic mouse line *Myh6-MerCreMer* that has been reported previously to target cardiomyocytes (Sohal et al. 2001). We administered the *Myh6-MerCreMer* + *;R26-tdTomato/* + mice with a dose of tamoxifen (0.2 mg/g of body weight) at P8W and harvested the hearts for analyses after 48 h. tdTomato were detected in cardiomyocytes, but not in cardiac fibroblasts, pericytes, endothelial cells, or SMCs (Fig. 4g). We noted that the intensity of tdTomato signals in cardiomyocytes were remarkably inhomogeneous; and the tdTomato signals in the cytoplasm were too weak to be identified in a significant part of cardiomyocytes (Fig. 4g). Therefore, the specificity of *Myh6-MerCreMer* in adult heart seems better than *Myh6-Cre* in this study, as *Myh6-Cre* is constitutively activated and also targets a few SMCs in adult hearts.

**Fig. 5:**
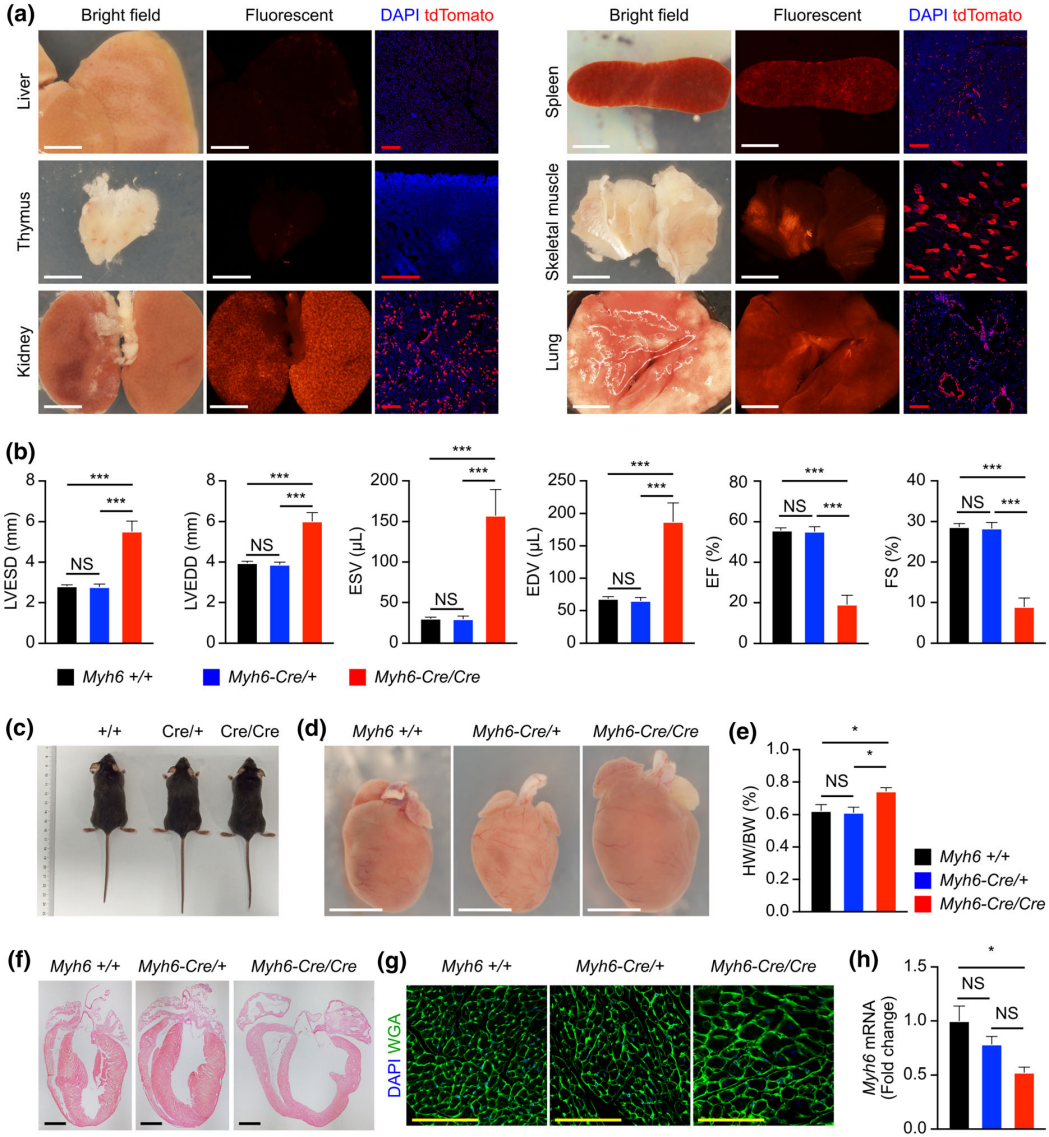
*Myh6-Cre* targets some other organs outside the heart and affects the expression of endogenous *Myh6* in heart. **a** tdTomato was detected in some other organs from the adult *Myh6-Cre/* + *;R26-tdTomato/* + mice at P8W. 3 mouse samples were examined. **b** ECG analyses of the P8W hearts with indicated genotypes. *n* = 6 mice per group. LVESD, left ventricular end-systolic dimension; LVEDD, left ventricular end-diastolic dimension; ESV, end-systolic volume; EDV, end-diastolic volume; EF, ejection fraction; FS, fractional shortening. **c** Whole bodies of the mice with indicated genotypes at P8W. **d** Whole-mount view of the hearts with indicated genotypes at P8W. **e** The relative heart weight was increased in the *Myh6-Cre/Cre* mice at P8W. HW, heart weight. BW, body weight. **f** HE staining shows the thinner left ventricular wall and ventricular septum in the hearts of *Myh6-Cre/Cre* mice at P8W. **g** WGA staining on heart sections. **h** The expression level of *Myh6* in the adult hearts at P8W. *n* = 3 mice per group in (**c**–**h**). White scale bars, 5 mm; Black scale bars, 2 mm; Red scale bars, 200 µm; Yellow scale bars, 100 µm. NS, non-significant; *p < 0.05; ***p < 0.001

Next, we examined whether *Myh6-Cre/* + *;R26-tdTomato/* + targets other tissues or organs outside the heart at P8W. Few tdTomato signals were detected in the liver or thymus, but plenty of tdTomato^+^ cells were identified in the kidney, spleen, skeletal muscle, and lung (Fig. 5a), suggesting the Cre expression in these non-cardiac tissues.

### *Myh6-Cre* affects the expression of endogenous *Myh6* in heart

*Myh6-Cre* strain can be maintained in the homozygous background and were also viable and fertile. However, we performed echocardiographic analysis at P8W and found that the heart functions of *Myh6-Cre/Cre* mice were compromised compared to wild type and *Myh6-Cre/* + groups (Supplementary Fig. S3, Fig. 5b). For instance, the left ventricular end-systolic dimension, left ventricular end-diastolic dimension, end-systolic volume, and end-diastolic volume were increased; and the ejection fraction and fractional shortening were decreased (Fig. 5b). There was no significant difference in heart function between the wild type and *Myh6-Cre/* + groups at P8W (Supplementary Fig. S3, Fig. 5b). The appearance of *Myh6-Cre/Cre* mice was normal (Fig. 5c), but the hearts were hypertrophic (Fig. 5d, e). Histological examination showed thinner ventricular septa and left ventricular walls in the hearts of *Myh6-Cre/Cre* mice (Fig. 5f). Wheat germ agglutinin (WGA) staining showed the cardiomyocyte size significantly increased in the hearts of *Myh6-Cre/Cre* mice (Fig. 5g). These data suggest that the homozygous *Myh6-Cre* strain exhibits cardiac hypertrophy at the adult stage. We also examined heart functions of *Myh6-Cre/* + mice at the later stage, and did not found any significant difference between the wild type and *Myh6-Cre/* + groups at postnatal 4 months (Supplementary Fig. S4).

Because the homozygous *Myh6-Cre* mice had deteriorative heart function, we speculated that the insertion of IRES-Cre-wpre-poly A cassette between the translational stop codon and the 3′ UTR may affect the expression of endogenous *Myh6* gene. Therefore, we performed real-time quantitative PCR and found that the expression level of *Myh6* was decreased in the hearts of adult *Myh6-Cre/Cre* mice compared with the wild type and *Myh6-Cre/* + groups (Fig. 5h). At the same time, the expression level of *Cre* was increased in the hearts of adult *Myh6-Cre/Cre* mice (Supplementary Fig. S5a). Western blot confirmed a reduced MYH6 protein level and increased Cre protein level in the hearts of adult *Myh6-Cre/Cre* mice (Supplementary Fig. S5b). The reason why *Myh6* expression is downregulated by the insertion needs further investigation.

### Deletion of desmoplakin gene in cardiomyocytes using *Myh6-Cre*

**Fig. 6:**
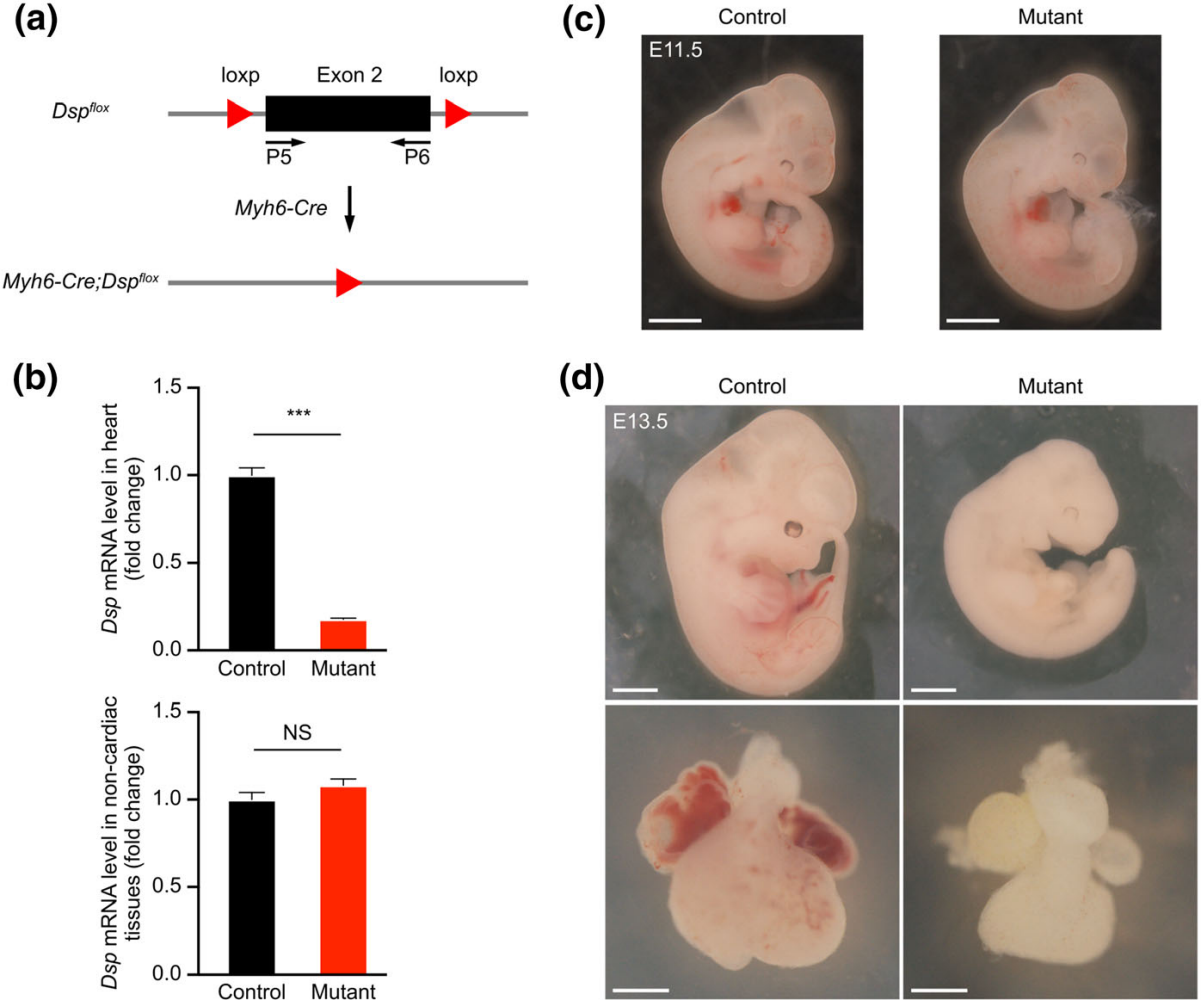
Inactivation of the *Dsp* gene in cardiomyocytes using *Myh6-Cre*. **a** The strategy for inactivating the *Dsp* gene in cardiomyocytes using *Myh6-Cre*. Primers P5 and P6 were used to detect exon 2. **b** The relative expression level of *Dsp* mRNA in hearts or non-cardiac tissues at E10.5. *** *p* < 0.001; NS, non-significant. *n* = 3 hearts per group. (**c**, **d**) Whole-mount view of embryos and hearts at E11.5 and E13.5. *n* = 4 embryos per group. Scale bars, 2 mm

Desmoplakin (Dsp) is one of the members of the desmosome gene family. To further examine the deletion efficiency of *Myh6-Cre*, we crossed *Dsp*^*flox*^ (*Dsp*^*fl*^) (Garcia-Gras et al. 2006) with *Myh6-Cre* mice in order to delete the Dsp gene in cardiomyocytes (Fig. 6a). We investigated whether *Myh6-Cre* was able to efficiently delete the Dsp gene in mouse hearts by crossing *Myh6-Cre/* + *;Dsp*^*fl/*+^ with *Dsp*^*fl/fl*^ mice. After this, we collected the embryos at E10.5 and performed real-time quantitative PCR experiments (primer P5 and P6 to amplify exon 2) to examine the mRNA levels of *Dsp* in the hearts (Fig. 6a). We found that *Dsp* mRNA was decreased in the hearts of mutant mice (*Myh6-Cre/* + *;Dsp*^*fl/fl*^) compared to their littermate controls (Fig. 6b). We also analyzed the *Dsp* mRNA in the embryos without hearts at E10.5, and did not find any significant difference between the mutant and control groups (Fig. 6b). These data suggested that the exon 2 of the Dsp gene was significantly deleted in the cardiomyocytes of mutant embryos.

Finally, we harvested the embryos at E11.5 and found that the morphology of the mutants was roughly normal compared to the controls (Fig. 6c). While at E13.5, the mutants appeared very pale and exhibited growth arrest at ~ E11.5. The mutant hearts did not beat and were significantly smaller than those of littermate controls at E13.5 (Fig. 6c), suggesting that the inactivation of the *Dsp* gene in the cardiomyocytes severely influences heart development and results in embryonic lethality during mid-term pregnancy.

## Discussion

A few constitutively activated Cre strains using *Myh6* promoter are commercially available, such as Tg(Myh6-Cre) (JAX stock #009,074) (Oka et al. 2006) and Tg(Myh6-Cre) (JAX stock #011,038) (Agah et al. 1997). These strains are transgenic and transgenes may not always correctly reflect the expression of endogenous *Myh6* temporally or spatially. For instance, the transgenic *Myh6-Cre* line was reported to begin excising at E9.5 (Agah et al. 1997; Gaussin et al. 2002; Xu et al. 2009; Papanicolaou et al. 2012), while the knockin line *Myh6-Cre* we generated in this study turns on as early as E8.5. Therefore, this *Myh6-Cre* knockin mouse line can be used to target cardiomyocytes since an earlier embryonic stage.

The *Myh6-Cre* in this study also labels a few SMCs in addition to cardiomyocytes in adult hearts. The inducible transgenic *Myh6-MerCreMer* strain (Sohal et al. 2001) is more specific in adult hearts likely because the MerCreMer allele can be activated in the restricted temporal window, while our *Myh6-Cre* is constitutively activated. Dynamic expression of Cre in unpredicted tissues or cells are always the issue for every constitutive Cre line.

Dsp is one of desmosomal genes, which also include desmocllin-2, desmoglein-2, plakophilin-2, and plakoglobin. Previous studies have identified mutations in all of the known desmosomal genes in patients with arrhythmogenic cardiomyopathy (Austin et al. 2019). Early embryonic lethality has been reported in the germline Dsp-null mice (Gallicano et al. 1998). Deletion of the Dsp gene in cardiomyocytes using transgenic *αMHC-Cre* mice also leads to high lethality in embryos (Garcia-Gras et al. 2006). The cardiac-restricted Dsp mutants exhibited growth arrest at E10-E12, and the specific phenotypes of the mutants have been investigated in more detail in the previous study (Garcia-Gras et al. 2006). In this study, just to examine the deletion efficiency of *Myh6-Cre*, we crossed the *Dsp*^*fl*^ mice with *Myh6-Cre* mice and found that the exon 2 of the Dsp gene was significantly removed in the E10.5 hearts by the *Myh6-Cre*. The Dsp mutants in this study exhibited growth arrest at ~ E11.5, in agreement with the previous observations (Garcia-Gras et al. 2006).

In summary, we established a new *Myh6-Cre* mouse model as an efficient tool enabling targeted gene deletion in cardiomyocytes. This model may thus significantly improve our knowledge and address critical questions regarding heart development and diseases.

## Supplementary Information

Below is the link to the electronic supplementary material.

Supplementary file1 (JPG 3091 KB)

Supplementary file2 (JPG 1133 KB)

Supplementary file3 (JPG 607 KB)

Supplementary file4 (JPG 853 KB)

Supplementary file5 (JPG 399 KB)

## Data Availability

All data generated and analyzed during this study are included in this published article.
