# Supplementary figures and images for: Generation and characterization of a Myh6-driven Cre knockin mouse line
Source: Transgenic Res. 2021 Sep 20;30(6):821–35. doi: 10.1007/s11248-021-00285-4 (PMC8580938; doi:10.1007/s11248-021-00285-4)

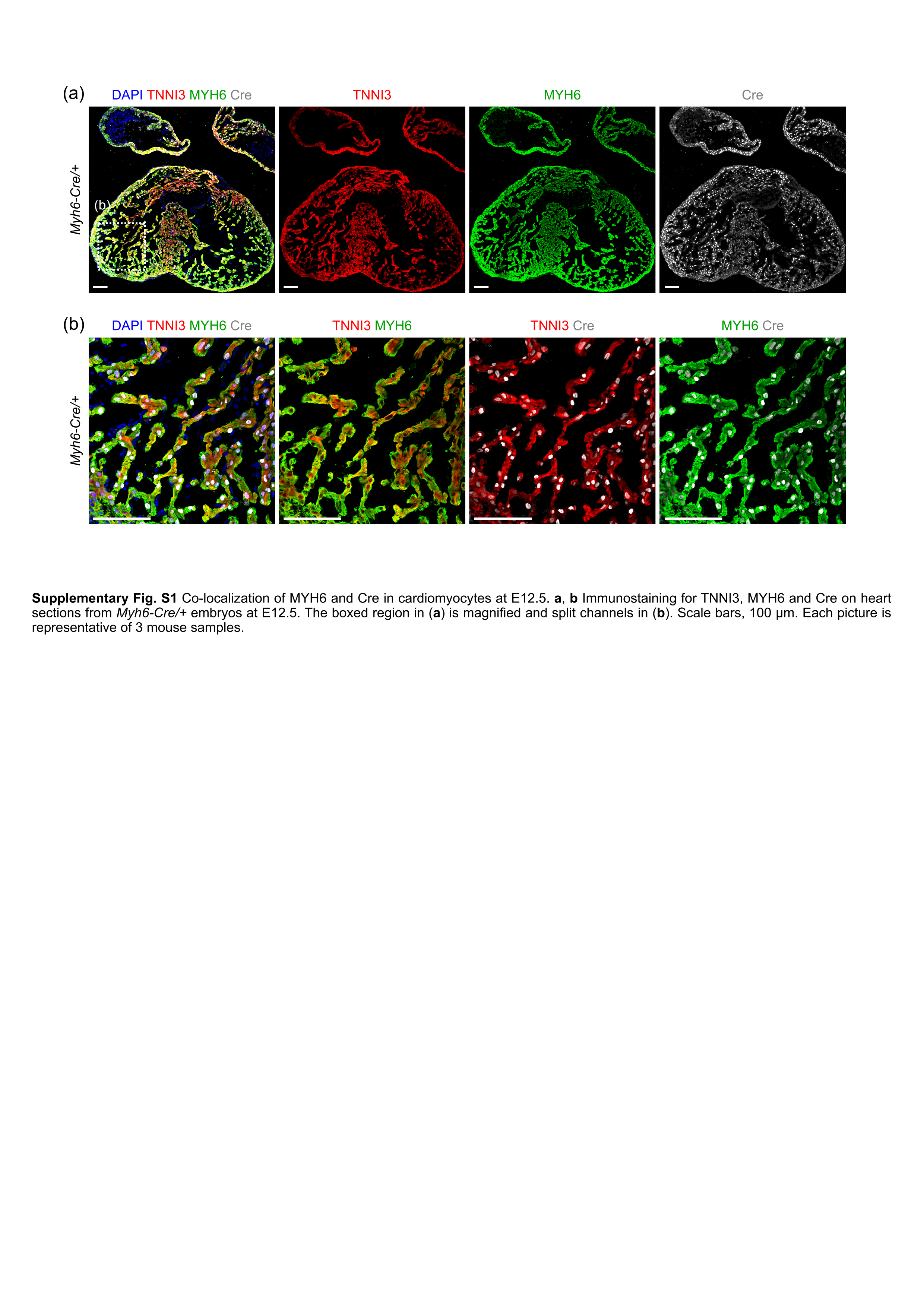

Supplement: Supplementary file 1 — Supplementary file1 (JPG 3091 KB) [file 11248_2021_285_MOESM1_ESM.jpg]

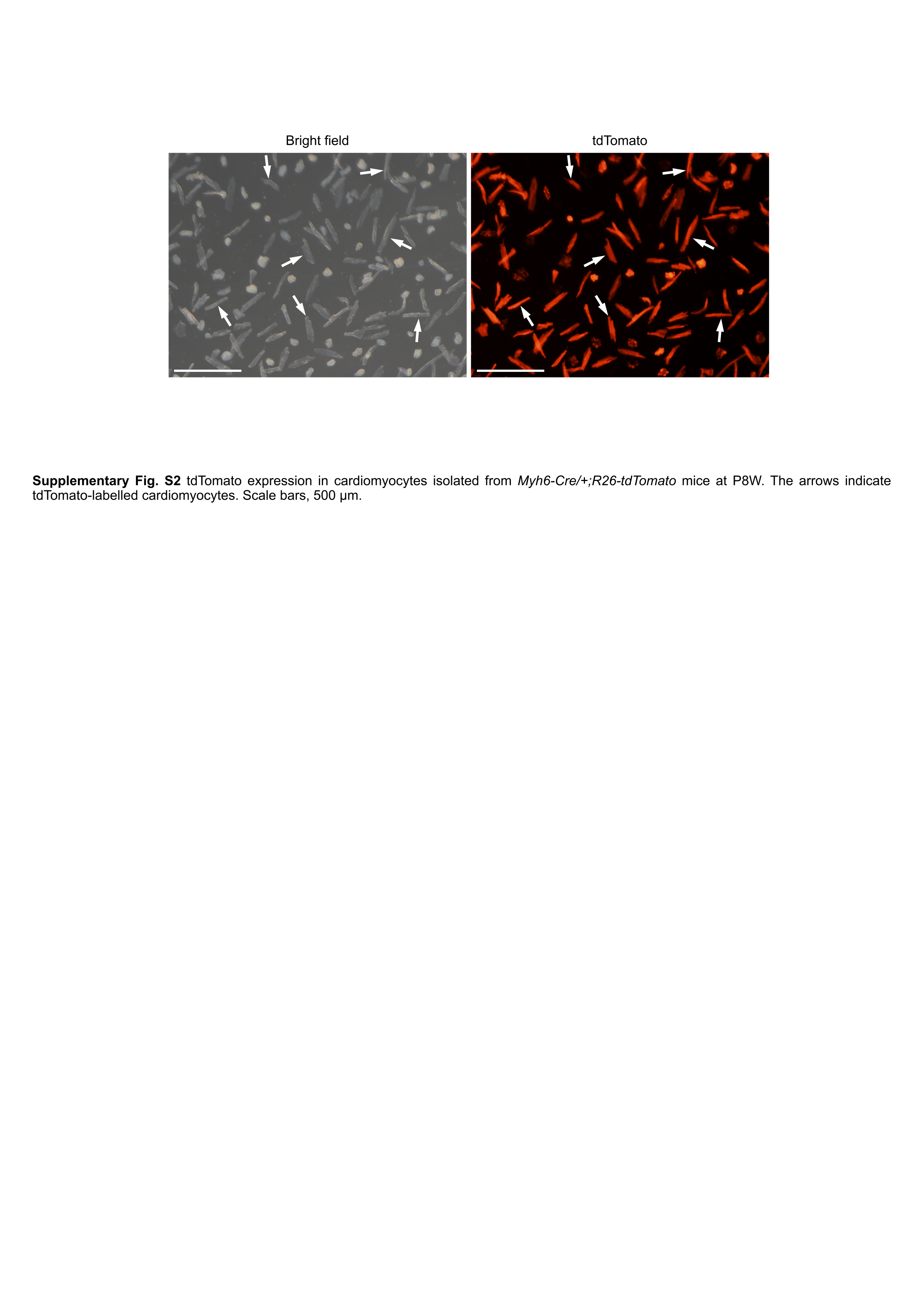

Supplement: Supplementary file 2 — Supplementary file2 (JPG 1133 KB) [file 11248_2021_285_MOESM2_ESM.jpg]

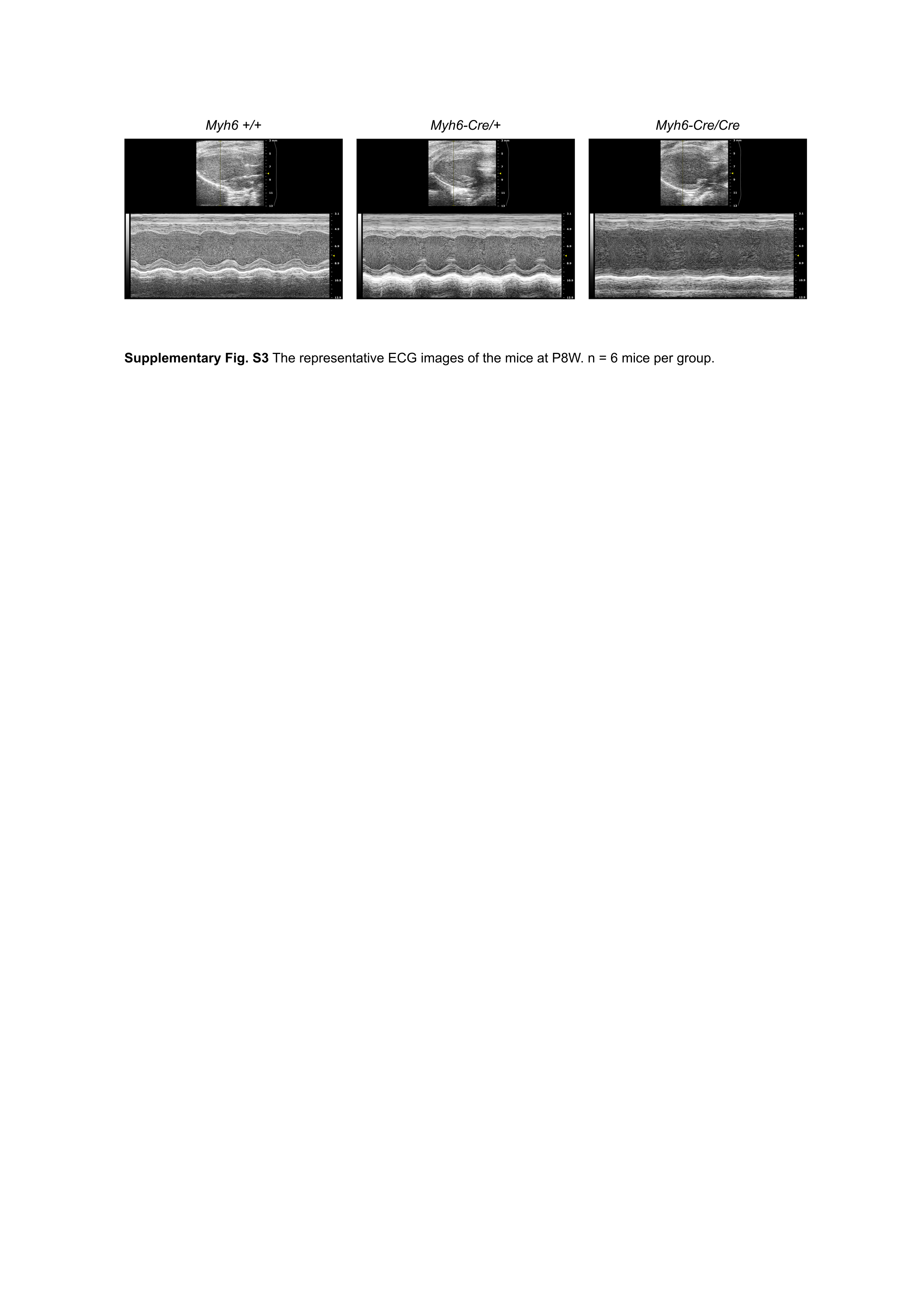

Supplement: Supplementary file 3 — Supplementary file3 (JPG 607 KB) [file 11248_2021_285_MOESM3_ESM.jpg]

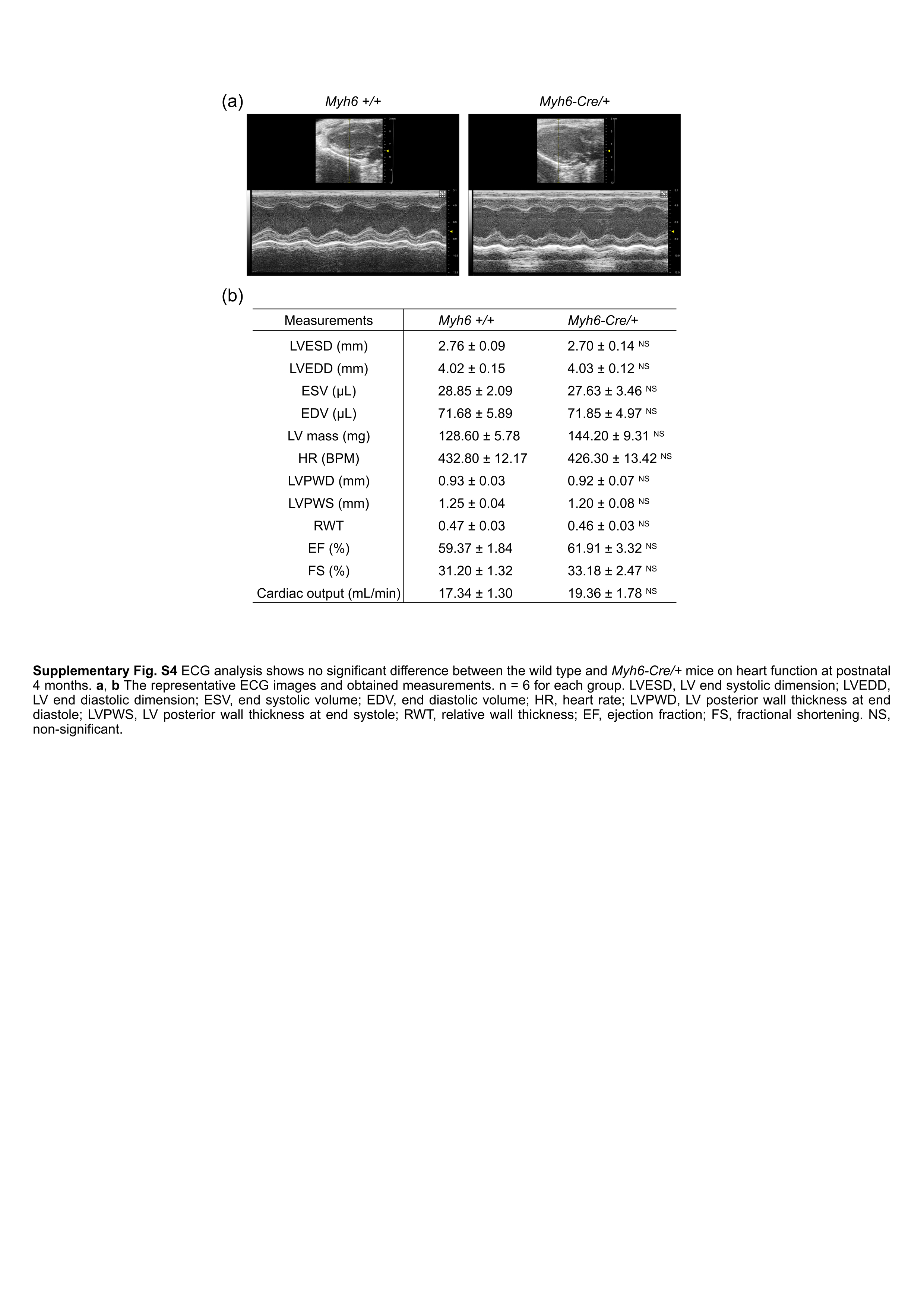

Supplement: Supplementary file 4 — Supplementary file4 (JPG 853 KB) [file 11248_2021_285_MOESM4_ESM.jpg]

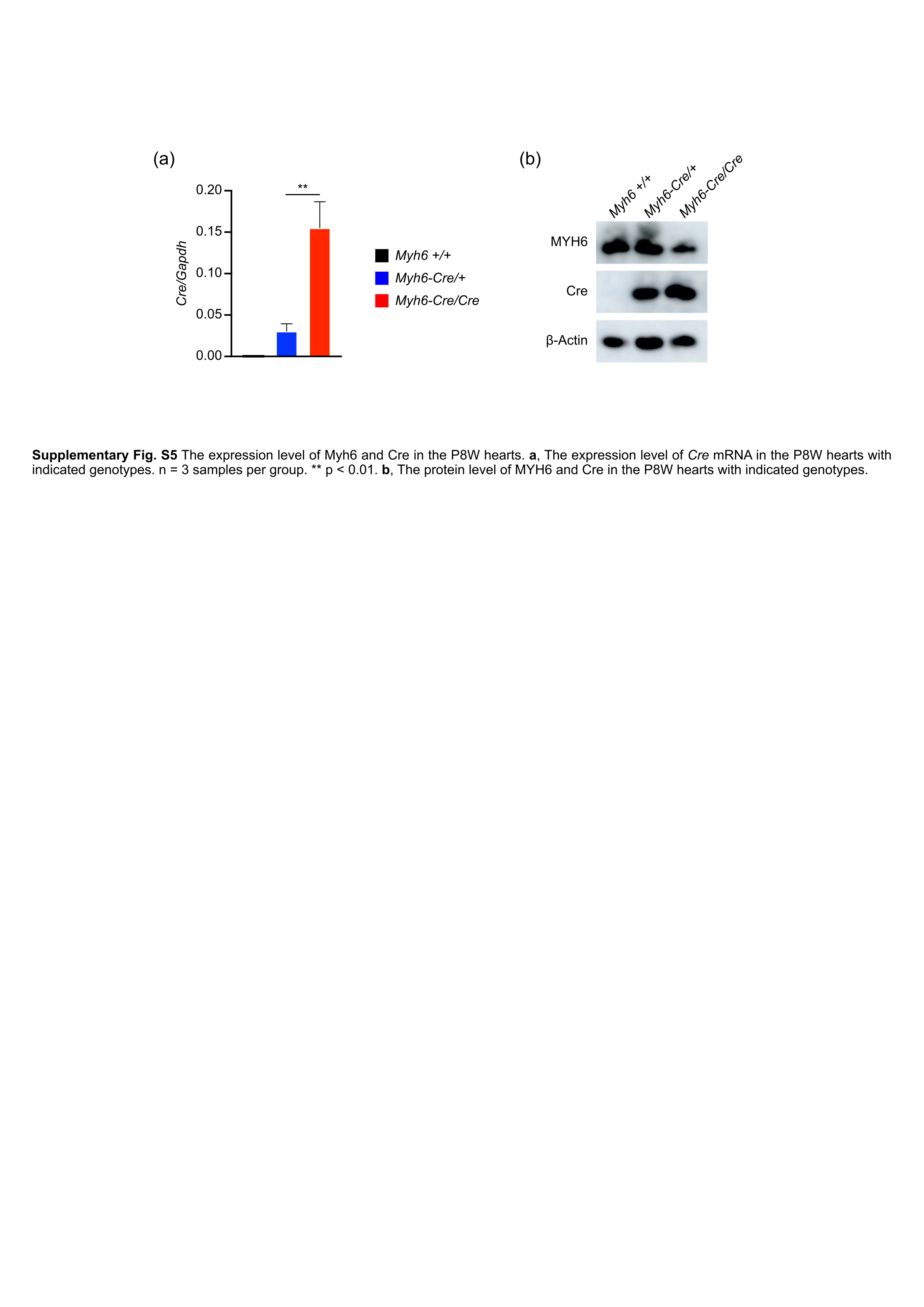

Supplement: Supplementary file 5 — Supplementary file5 (JPG 399 KB) [file 11248_2021_285_MOESM5_ESM.jpg]
